# The Effect of Microbial Inoculum and Urea Supplements on Nutritive Value, Amino Acids Profile, Aerobic Stability and Digestibility of Wheat and Corn Silages

**DOI:** 10.3390/ani13132197

**Published:** 2023-07-04

**Authors:** Philip Wagali, Ira Pelech, Chris Sabastian, Julius Ben Ari, Haim Tagari, Sameer J. Mabjeesh

**Affiliations:** 1 Department of Animal Sciences, The Robert H. Smith Faculty of Agriculture and Environment, The Hebrew University of Jerusalem, Rehovot 7610001, Israel; philip.wagali@mail.huji.ac.il (P.W.); ira.pelech@mail.huji.ac.il (I.P.); chris.sabastian@mail.huji.ac.il (C.S.); haim.tagari@mail.huji.ac.il (H.T.); 2 The Laboratory for the Mass Spectrometry and Chromatography, Interdepartmental Analytical Unit (ZABAM), The Robert H. Smith Faculty of Agriculture and Environment, The Hebrew University of Jerusalem, Rehovot 7610001, Israel; masstocharge@gmail.com

**Keywords:** silage, corn, wheat, microbial inoculum, amino acids, digestibility

## Abstract

**Simple Summary:**

Wheat and corn silages are common feedstuffs used in Israel. For long-term use of the feedstuffs without compromising the nutritional value, there is a need for an effective preservation method. We conducted a study to assess the effect of adding microbial inoculum (MI) and urea on the chemical composition including the amino acids profile, aerobic stability and in vitro digestibility of wheat and corn silages. The silages were subjected to four treatments: control, MI, urea and a combination of MI + urea. Silages were analyzed for quality parameters and in vitro digestibility. The results showed that additives improved the quality parameters of wheat and corn silages. The inclusion of MI produced the most aerobically stable silages, whereas the inclusion of urea compromised its aerobic stability. None of the additives affected the true CP content of silages. Additives improved in vitro cell wall carbohydrates’ digestibility in both silages and had the best production when MI was combined with urea. These results imply that additives enhance the nutritional value, aerobic stability and digestibility of silages.

**Abstract:**

Wheat and corn silages are widely used as ruminant feed in Israel due to their availability and cost-effectiveness. To ensure long-term preservation without compromising nutritional quality, effective methods must be employed. The inclusion of additives during harvest and ensiling can enhance efficiency and address preservation challenges. In the current study, the effects of microbial inoculum (MI) and urea on the chemical composition, amino acid profiles, aerobic stability, and in vitro digestibility of wheat and corn silages were investigated. Samples of wheat and corn were subjected to four treatments: control, MI, urea and a combination of MI + urea. The treatments were ensiled in anaerobic conditions and opened after 1, 7, 14 or 28 days. The results showed that additives improved the quality parameters of wheat and corn silages. The inclusion of MI produced the most aerobically stable silages. The inclusion of urea in silages decreased aerobic stability. Additives improved in vitro cell wall carbohydrates’ digestibility in both silages and was the best when MI was combined with urea. These results imply that additives could be incorporated in silages to enhance their nutritional value, aerobic stability and digestibility. Nonetheless, increased CP content with additives was not accompanied with a parallel increase in amino acids’ content in corn silage.

## 1. Introduction

Ensiling is one of the best known successful preservation methods with minimal nutritional value loss [1]. This feed preservation method is dependent on several factors: the first important one before ensiling is the stage of maturity at which the silage material is harvested. The anaerobic ensiling conditions facilitate lactic acid production by lactic acid bacteria (LAB) that may occasionally be accompanied by other organic acids such as acetic acid. These acids are responsible for the decrease in pH and for stabilizing the silage [2]. The contribution of short chain volatile fatty acids (VFAs; acetic, propionic and butyric acids) to silage acidity is negligible because of them being weak acids and their relatively low concentration in silages. However, every acid has a unique organoleptic feature, which may be positive or negative in silages. For instance, acetic acid improves the aerobic stability of silages and propionic acid is implicated in improving the flavor of the silage, whereas butyric acid is usually associated with a bad odor indicative of the activity of undesirable microorganisms during ensiling [3]. A rapid production of these acids minimizes nutrient loss. Moreover, the chopping mechanism and dry matter (DM) content of ensiled material, and the transportation, particle size and sugars as well as any other supplements will influence the fermentation process and the quality of the silage [1,4].

A wide range of ensiling additives are available on the market. The goals of these are mainly to reduce DM losses during the ensiling process, encourage a rapid drop in pH value, prolong the shelf life during aerobic exposure after opening the bunker/silo and to increase intake by ruminants [5]. Biological additives such as anaerobic bacteria inoculum are preferred to chemical additives [6].

The use of heterofermentative microbial inoculum (MI), also known as effective microorganisms (EM), is a technology that was developed by Professor Teruo Higa at the University of Ryukyus, Okinawa, Japan, in 1982 [7,8]. This MI is a liquid preparation that contains beneficial bacteria (including LAB) in the form of yoghurt, grown on a medium of sugar cane molasses and water [7]. Microbial inoculum operates on the principle of competitive exclusion [9]. Bacteria produced in MI secrete materials that support life, health and rejuvenation including: vitamins, enzymes, antioxidants, amino acids, etc., according to [8,10]. By doing so, they create in their environment a positive and powerful presence that makes it difficult for unfavorable microorganisms to reside and thrive [10,11,12].

On the other hand, an additive such as urea provides available N to microbes during the ensiling process and hence may prevent proteolysis of plant material and may increase microbial protein [1]. Moreover, excess urea-N provides non-protein nitrogen (NPN), which is beneficial in ruminants’ rations since it is a major building block in the synthesis of amino acids and proteins by ruminal bacteria [13,14]. However, in order to be efficiently utilized, urea should be added to high available energy forages with relatively low protein content such as corn in order to obtain an optimal synchrony between available energy and N. However, the limitation of using urea as a silage additive is that it impairs the decline in pH, which is vital in terms of preserving the silage [14].

Corn and wheat are the major fodder crops in Israel used for silage making. Corn is used for making silage in summer and wheat is used for making silage in winter, allowing a continues supply of high-quality forage resources for high-producing dairy cows’ rations. To overcome challenges such as climate changes, aerobic stability after opening the bunker, minimizing DM losses and improving nutritional values, additives including MI and urea might be beneficial [15].

We hypothesized that the addition of MI and urea (combined or separately) would improve the ensiling process by enhancing the rapid decrease in pH and maintaining it, improve aerobic stability and improve the digestibility of wheat and corn silages. We also hypothesized that urea might increase the CP content and alter the AAs profile by minimizing the degradation of plants’ N and enhancing bacterial protein synthesis.

## 2. Materials and Methods

### 2.1. Experimental Design

Prior to ensiling the forages, one-liter glass jars (equipped with a rubber band and lid that enable gas release only; Foxhome, Rehovot, Israel) were sterilized in an oven at a temperature of 120 °C for 48 h to kill any undesirable bacteria that would interfere with the ensiling process. The jars were packed with chopped corn forage, airtight sealed to simulate the anaerobic conditions in the silo. Following the challenges with the jars (tedious and difficult compaction), vacuum bags were used as a more viable and efficient option for ensiling forages. The method was approved in our laboratory as efficient as the glass jars and was used for preparing wheat silage as described below.

The wheat silages were packed into nylon bags and vacuum sealed using a vacuum machine (Kunba; model DZ-400/ZT, Kesem, Israel). The sealing of the vacuum bags was easier and faster, generated a 100% airtight environment and most importantly created uniformity in the compression of the ensiled material.

Wheat and corn forages were harvested at the right maturity period during the growing season from commercial fields that were meant to be ensiled. The harvested corn was collected at the end of August, in Nahalal, Israel (32°41′24″ N, 35°11′48″ E), while the harvested wheat was collected at the beginning of April, in Masu’ot Yitzhak, Israel (31°42′12″ N, 34°41′22″ E). As part of the harvest process, the forage was mechanically chopped to a length of 2–4 cm prior to ensiling.

The corn and wheat forages were divided into four treatments before ensiling. Control, where corn or wheat forage was ensiled without any additives. MI, a liquid microbial inoculum additive (supplied by EM-Zoo^®^, Aseret, Israel), which was applied at a dosage of 1 L per 1 ton of fresh matter according to the manufacturer’s instructions. Urea, an addition of 4 L of liquid urea with a concentration of 21% N, which was applied at a rate of 4 L per 1 ton of fresh matter. MI + urea, a combined treatment comprising both preparations, with the same dosages detailed as above.

Each treatment was mixed separately in a 63 L electric concrete mixer (Karnaf, HCM650, Petah Tikva, Israel) for 10 min in order to homogenously mix the additives. This was followed by packing corn forage in glass jars and wheat in vacuum-sealed bags. Both the jars and vacuum-sealed bags were weighed and marked before forage packing. Approximately 1 kg of fresh matter was packed in the jars or vacuum-sealed bags. Weight after packing was also recorded. All treatments were ensiled at room temperature for 1, 7, 14 and 28 days (n = 4 for each ensiling period). The 28 day period was chosen because fermentation often stabilizes between 7 and 14 days in controlled conditions.

### 2.2. Chemical Analyses of Silages

After opening the jars or vacuum-sealed bags, 100 g of silage was sampled and immediately agitated with 400 mL of distilled water in a blender (Vitamix, model E310, Natanya, Israel). The filtrate was used for pH measurement using a pH meter (Satorius Ag-Gottingen, Germany). A portion of the filtrate was used for microbial analysis, enumeration of LAB using Rogosa SL Agar (Himedia, Mumbai, India) according to [16], enumeration of molds and yeast using Malt Extract Agar (Sigma-Aldrich, Rehovot, Israel) according to the detailed method described by [17]. The colonies were counted using a Colony Counter device (CLC-57, MRC, Holon, Israel). Other portions of the filtrate were stored at −20 °C and were later used for VFAs’ analysis [18] using *hp* gas chromatograph (model 5890A), lactic acid (LA) concentration using a calorimetric method [19].

The rest of silage samples after opening (day 1, 7, 14 and 28) were dried at 60 °C for 48 h, ground through a knife mill (Thomas-Willey Laboratory Mill, model 4, Philadelphia, PA, USA) to pass through a 2 mm screen. Samples were subjected to full chemical analysis. Absolute dry matter (DM) was determined by placing samples in an air-forced oven at 105 °C overnight. Ash and organic matter (OM) were determined by ashing in a muffle furnace at 600 °C for 3 h. Ether extract (EE) was determined using the Soxhlet method with petroleum ether (30–40 °C; Merck, Rehovot, Israel) as solvent for 8 h. Crude protein content was measured using the Kjeldahl method with an automatic Kjeldahl machine (KjelMaster K-375-Buchi, Flawil, Switzerland).

Neutral detergent fiber (NDF; heat stable alpha-amylase was added to solution), acid detergent fiber (ADF) and hemicellulose analyses were also performed using an Ankom machine (Ankom^220^ Fiber Analyser^®^_,_ Macedon, NY, USA) as described by [20]. In vitro dry matter digestibility (IVDMD) and in vitro NDF digestibility (IVNDFD) assays were performed according to the protocol of [21]. Rumen fluid was withdrawn from two ruminally fistulated wether Assaf sheep that were maintained on standard ration containing 2.42 mega calories of metabolic energy, 12% CP per kg DM basis. Ration consisted 73% roughage feeds (wheat silage, clover hay, wheat hay), and the rest grains, minerals and vitamins to satisfy the maintenance requirements according to NRC recommendations [22]. The use and procedures for the fistulated sheep were approved by the IACUC (AG-15544), in accordance with the Animal Safety Guidelines of the Hebrew University of Jerusalem.

Dry samples of silage materials of the 28 day period were subjected to acid hydrolysis (n = 4) to determine amino acids’ (AAs’) content after grinding to pass a 1 mm screen according to detailed protocol described by [23] including recovery test. The quantitative analysis of AAs was carried out using the LC-MS/MS system, which consisted of Nexera X2 UPLC (Shimadzu; Laval, QC, Canada) coupled to the QTRAP 6500+ mass spectrometer (Sciex; Toronto, ON, Canada). Chromatographic separations were carried out using HILC-Z HPLC column (150 × 2.1 mm, 2.7 µm, Agilent) employing linear gradient of acetonitrile/water with 100 m*M* ammonium formate. The mass spectrometer was operated upon positive ESI in MRM mode. Calibration samples containing 28 individual amino acids (purchased from Sigma-Aldrich; Rehovot, Israel) were prepared at concentrations from 5 ng/mL to 5000 ng/mL. The samples were spiked with a mixture of 20 isotopically labeled AAs used as internal standards (purchased from Cambridge Isotopes; UK) at final concentration 100 ng/mL per sample. AAs profile was expressed as g/100 CP.

### 2.3. Aerobic Stability Assay

Samples (approximately 150 g) for ensiling days 14 and 28 were incubated for 5 days at room temperature as described by [24]. At the end of the 5th day of aerobic exposure, the amount of CO_2_ emitted was calculated and later was used to calculate sugar loss. Samples of the 28 days silage were used for pH measurement and molds and yeast enumeration.

### 2.4. Statistical Analysis

Data were exported from an excel sheet to JMP Pro^®^ (Ver. 16.0.0, SAS Institute Inc., Cary, NC, USA) and subjected to a two-way ANOVA (additive, day of ensiling and interaction). The data were further subjected to Dunnett’s test to compare the control to the additive treatments at *p* < 0.05. The data are presented as LSMeans and SEM.

## 3. Results

The chemical composition of the corn and wheat forages prior to ensiling is summarized in Table 1. The dry matter content of wheat forage was 31%, while it was 44% for the corn vegetative material. The pH values of the wheat and corn forages were 5.5 and 6.5, respectively. The crude protein content was higher in the corn plant material as expected compared to the wheat material. Corn forage had some advantage with regard to the hemicellulose content.

### 3.1. pH of Wheat and Corn Silage

The results of wheat silage pH showed that there was an interaction effect between treatment and days of ensiling (*p* < 0.0001; Table 2). The main reason for this was the inconsistent changes (drop) in the pH values between the days and treatments effects. The results also demonstrate that a steep drop in pH happened between days 1 and 7 and milder changes continued for 28 days of ensiling (*p* < 0.0001). On average, the lowest pH values were recorded in the MI treatment followed by the control, urea and MI + urea. At 28 days of ensiling, pH values differed from the control (3.84), with the lowest being for MI (3.73), intermediate levels for MI + urea (3.90) and the highest for urea (3.93).

Looking at the corn silage values (Table 2), there was an interaction between the treatment and days of ensiling (*p* < 0.0047). This mainly happened because changes in the pH values in the MI treatment showed a mild increase up to 28 days (3.7 at day 7 vs. 3.9 at day 28) in contrast to the rest of the treatments, which showed a similar value to that at day 7. Similar to wheat silage, the main drop in pH values happened between days 1 and 7; however, bear in mind that the values of the original material were dramatically different (Table 1). At day 28, compared to the control, pH values were highest in the MI treatment (3.97 vs. 3.68).

### 3.2. Temperature of Wheat and Corn Silages

**Figure 1:**
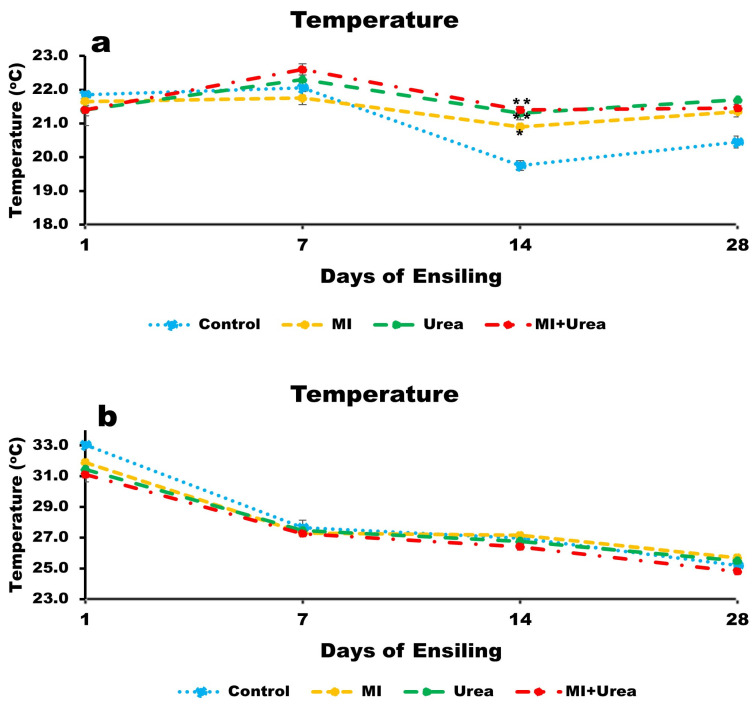
(**a**) Temperature of wheat silage with additive treatments at various days of ensiling; (**b**) temperature of corn silage with additive treatments at various days of ensiling. * 0.05 and ** 0.01: statistical significance after LSMeans Dunnett test.

The temperature of wheat silage was different among treatments: lowest in the control and highest in urea and MI + urea (*p* < 0.0087; 21.0, 21.4 and 21.7 °C for control, MI and both urea and MI + urea, respectively; Figure 1a). The temperature was also different among the days of ensiling with the highest being at day 7 and the lowest at day 14 (*p* < 0.0001; 21.6, 22.2, 20.8 and 21.2 °C for days; 1, 7, 14 and 28, respectively). Interaction between treatment and days of ensiling (*p* < 0.0292) existed, mainly because of the behaviour of the temperature drop in the control compared to the rest (Figure 1a). It is worth noting that the temperature differences in wheat silage were less than 1 °C.

Corn silage had an inverse trend for treatment; the highest temperature was recorded in the control and the lowest in MI + urea (*p* < 0.0001; 27.4, 27.8, 28.0 and 28.2 °C for MI + urea, urea, MI and the control, respectively; Figure 1b. However, days of ensiling had a trend similar to wheat silages with the highest temperature recorded on day 1 and the lowest on day 28 (*p* < 0.0037; 27.4, 26.8 and 25.3 °C for 1, 7, 14 and 28 days, respectively). It is also worth mentioning that the temperature was between 3 and 10 °C higher in corn than in wheat silages (seasonal differences).

### 3.3. In Vitro Digestibility of Wheat and Corn Silage

#### 3.3.1. In Vitro DMD of Wheat and Corn Silage

An interaction of day and treatment effect existed on the IVDMD of wheat silage. Mainly, this effect correlated to the behaviour of the IVDMD of the control compared to the rest of the treatments (Table 3). In the control treatment, the IVDMD increased by time (57.2 to 61.7%), while the rest of the treatments decreased by days of ensiling. However, at 28 days of ensiling, the urea treatment was the lowest compared to the control and other treatments (52.6 vs. 57.6%). The main effect of treatment was significant on the IVDMD and appeared to be the highest in the control and the lowest for urea and MI + urea, and intermediate for MI (*p* < 0.0058; 58.4, 56.7, 55.1 and 54.8% for the control, MI, MI + urea and urea, respectively; Table 3). Days in ensiling did not affect the IVDMD.

For corn silage, on the other hand, IVDMD was affected by the treatment, being highest in the control and MI and lowest for urea and MI + urea (*p* < 0.006; 58.4 vs. 49.2%; Table 3). At day 28 of ensiling, the urea treatment differed from the control and was lowest among the treatments (45.9 vs. 54.1%).

#### 3.3.2. In Vitro NDFD of Wheat and Corn Silage

The interaction effect of treatment by days of ensiling was observed on IVNDFD in wheat silage. This existed because in general, days negatively affected IVNDFD; however, this was only true during all days in MI treatment. The drop in IVNDFD was not consistent with the rest of the treatments compared to MI. For example, in MI + urea, IVNDFD dropped down until day 14 and then increased toward 28 days (42% vs. 45%). In the urea treatment, between day 7 and 14 there was an increase, and then a steep drop toward day 28 (42% vs. 44% vs. 40%, respectively). Additives increased IVNDFD in wheat silage with the highest values measured in MI and the lowest in the control treatment (*p* < 0.0001; 44.5, 43.9, 42.2 and 40.1% for MI, MI + urea, urea and control, respectively; Table 4). IVNDFD decreased with the increase in ensiling days (*p* < 0.0014; 44.0, 42.6, 42.4 and 41.6% for days 1, 7, 14 and 28, respectively. At 28 days in ensiling, IVNDFD was highest in MI + urea compared to the control and the rest of the treatments.

Similar to wheat silage, corn silage exhibited interaction effects of treatment by days of ensiling on IVNDFD (Table 4). This existed because of the inconsistency of treatment effects during the ensiling period. For example, MI + urea and urea almost did not affect the values of IVNDFD over time. However, in the control and MI treatments, there was a decrease in the values, especially after day 7 through 28. Nonetheless, the main effect of treatment revealed the highest values were for MI + urea and urea (47%) compared to lower values (40%) in the control and MI. Corn silage IVNDFD generally decreased with days of ensiling. It was highest at day 7 and lowest at day 14 (*p* < 0.0368; 44.8, 43.6, 43.4 and 42.5% for days 7, 1, 28 and 14, respectively). At day 28 of ensiling, the MI + urea and urea treatments were the highest compared to the control. Compared to control values, the IVNDFD levels of corn silages were the highest in MI + urea through all the days.

### 3.4. Volatile Fatty Acids’ Concentration of Wheat and Corn Silage

Table 5 describes the concentration of individual and total VFA in wheat silage. Total VFA did not differ between treatments and averaged 2.16 g/100 g DM. However, propionic acid concentration decreased with days of ensiling and stabilized after day 14 (Table 5). Butyric acid could barely be detected.

For corn silage, additives treatment and days in ensiling increased ethanol concentration (Table 6). It was highest in MI + urea compared to the other treatments (*p* < 0.0001; 10.50, 5.12, 3.98 and 3.61 g/100 g DM for control, MI + urea, urea and MI, respectively; Table 6).

Total VFA production in corn silage was affected by treatment and days of ensiling, being the highest at day 28 and the lowest at day 7 (*p* < 0.0001; 3.53, 2.94, 2.49 and 2.14 g/100 g DM for day 28, 14, 7 and 1, respectively). However, at 28 days, concentrations of total VFA were similar among treatments. It is worth mentioning that individual VFAs such as propionic and acetic acids were affected by treatments and days of ensiling (Table 6) and there was a noticeable increase by days. Nonetheless, at day 28 of ensiling, acetic acid concentration was similar for all treatments and averaged 3.21 g/100 g DM, and propionic acid was highest in the MI treatment. An interaction effect existed on propionic acid because in the MI treatment compared to others, it continued to increase, while in the rest, it showed a constant concentration.

### 3.5. Lactic Acid Concentration of Wheat and Corn Silage

**Figure 2:**
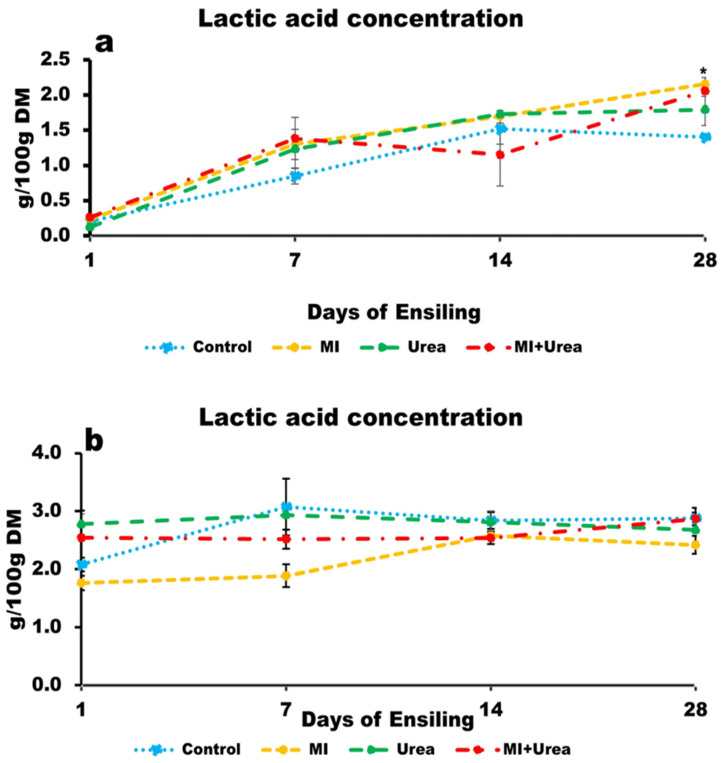
(**a**) Lactic acid concentration of wheat silage with additive treatments at various days of ensiling; (**b**) Lactic acid concentration of corn silage with additive treatments at various days of ensiling. * 0.05 statistical significance after LSMeans Dunnett test.

The concentration of LA in wheat silage tended to be statistically different for additive treatment; the additives increased LA: the control had the lowest and MI had the highest concentrations (*p* < 0.0772; 1.0, 1.2 and 1.3 g/100 g DM for control, MI + urea, urea and MI, respectively; Figure 2a). LA increased with ensiling days (*p* < 0.0001; 0.2, 1.2, 1.5 and 1.9 for days; 1, 7, 14 and 28, respectively).

For corn silage, LA concentration was lowest in the MI and highest in the urea additive treatments (*p* < 0.0084; 2.2, 2.6, 2.7 and 2.8 for MI, MI + urea, control and urea, respectively). Corn silage also tended to be statistically different for ensiling days: it increased with ensiling days with the lowest concentration at day 1 and the highest at both days 14 and 28 (*p* < 0.0769; 2.3, 2.6 and 2.7 g/100 g DM for 1, 7 and both days 14 and 28, respectively; Figure 2b).

### 3.6. Aerobic Stability of Wheat and Corn Silage

**Figure 3:**
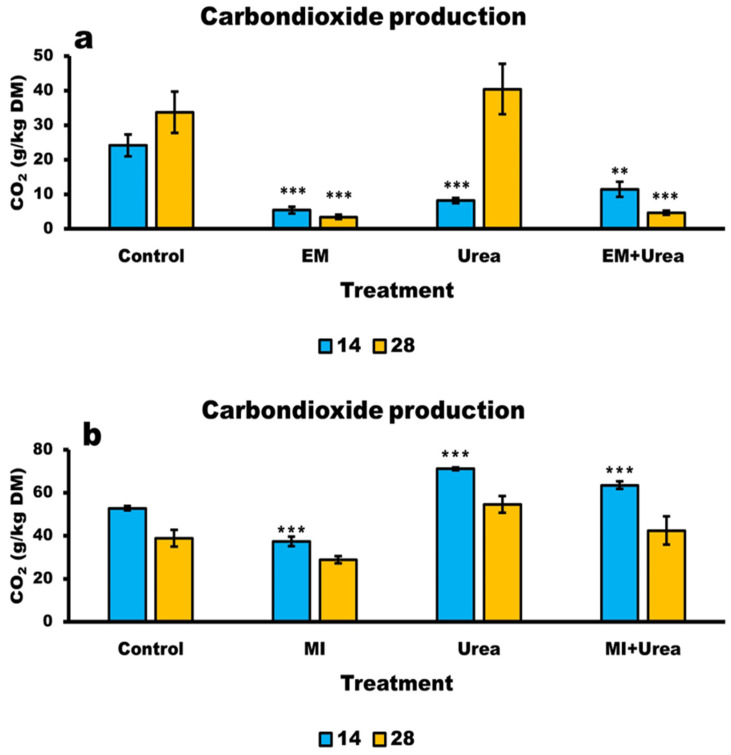
(**a**) The amount of carbon dioxide production from wheat silages during a 5-day exposure to air at 14 and 28 days of ensiling; (**b**) The amount of carbon dioxide production from corn silages during a 5-day exposure to air at 14 and 28 days of ensiling. ** 0.01 and *** 0.001: statistical significance LSMeans Dunnett test.

Additives dramatically reduced CO_2_ production in wheat silage. CO_2_ production was highest in the control (more than 7-fold) compared with MI (Table 7). CO_2_ emissions increased almost twice with ensiling days (*p* < 0.0217; 20.5 and 12.3 g/kg DM for days 28 and 14, respectively). CO_2_ production was significantly different between treatments and day of ensiling, with a lower production on day 14 than day 28 (*p* < 0.0011; Figure 3a and Table 7). The interaction effect that existed can be explained by the behaviour of CO_2_ release from the control and MI treatments, which was linear between days 14 and 28, while in MI + urea and urea, it was flat (Figure 3a). The calculated sugar loss equivalent also followed a similar trend to that of the CO_2_ production for additive treatments. At day 28, the aerobic exposure caused a significant loss of sugars in the control and urea treatments (25.2 g) compared to MI and MI + urea (2.75 g). Molds count levels and pH values after 5 days of aerobic exposure at 28 days of ensiling (Table 7) for all treatment additives were significantly smaller than those of the control. The pH values of the exposed silage with additives were on average 4.37 compared to 7.1 in the control and lowest (3.9) for the MI treatment.

The aerobic exposure of corn silage showed a different phenomenon than the wheat silage (Table 7 and Figure 3). CO_2_ production was the lowest for MI at 28 days of ensiling and all treatments differed from the control, with the highest for MI + urea. Days in ensiling decreased CO_2_ production (*p* < 0.0001), and at 28 days in silage, all treatments had a similar value (41.1 g/kgDM). The same trends were observed for the calculated sugar losses. Molds and pH did not differ between treatments after exposure for 5 days in corn silage at 28 days of ensiling, and averaged 9.2 (Log CFU/gDM) and 4.0, respectively.

### 3.7. Chemical Composition of Wheat and Corn Silages

#### 3.7.1. Crude Protein Content of Wheat and Corn Silages

The crude protein content of wheat silages was different among treatments, with the lowest content in the control and MI, and the highest in urea and MI + urea (*p* < 0.0005; 9.0, 9.2, 9.6 and 9.7% for the control, MI, urea and MI + urea, respectively; Table 8). At days 1 and 7, both urea and MI + urea were higher in CP content compared to the control. However, at day 28, all treatments were similar.

An interaction effect of treatment by days of ensiling existed on CP content in corn silage (Table 8). This interaction was mainly caused by the different behaviours within the treatments of the ensiling process. For example, both treatments involving the addition of urea began with a higher CP content, and while in the urea treatment the CP content was constant during the days of ensiling, in MI + urea, the CP content increased at day 28 to reach 12.5%. On the other hand, in the control treatment, the CP content increased up to day 14 and then decreased at 28 days of ensiling. In MI treatment, the CP content increased by days of ensiling and reached 10.5% at day 28 compared to 8.35% at day 1. In general, the results for corn silage showed that CP was different among treatments, and was lowest in the control and highest in MI + urea (*p* < 0.0001; 8.4, 9.5, 11.0 and 11.1% for the control, MI, urea and MI + urea, respectively). CP content had statistical differences among days of ensiling with the lowest content at day 1 and the highest at day 28 (*p* < 0.0001; 9.5, 9.9, 10.1 and 10.5% for days; 1, 7, 14 and 28, respectively). Compared to the control, all treatments were higher in CP content during the ensiling days.

#### 3.7.2. Cell Wall Carbohydrates and Other Parameters of Wheat and Corn Silages

All detailed data on the cell wall carbohydrate content of both silages (NDF, ADF, hemicellulose) and DM content are presented in the Supplementary Material; Figure S1 and Tables S1 and S2. In general, wheat silage differed in NDF and ADF among treatments with the highest value in the control compared with the rest (*p* < 0.005; 59.4 vs. 58.0% and 29.2 vs. 28.2%, respectively; Figure S1). Days of ensiling caused an increase in NDF and ADF content and was lowest at day 1 (*p* < 0.0001; 56.4 vs. 59.1%, and 27.6 vs. 28.8%, respectively; Figure S1). Hemicellulose content was similar (*p* < 0.07) among treatments and averaged 29.7%; Table S1. However, during the ensiling process, hemicellulose content increased up to day 7 and then decreased to reach the values of day 1 (Table S1). DM content of silages at 28 days of ensiling was 42.7% and decreased during ensiling (Table S2).

Corn silage NDF, ADF, hemicellulose and DM contents differed between treatments (Figure S1 and Tables S1 and S2). NDF content was lower in the urea treatment compared to the rest (*p* < 0.0001; 48.4 vs. 51.6%). During ensiling, NDF content interchanged and was highest at day 15 (*p* < 0.0002; 52.8%), and then at 28 days it stabilized to be 50.1%. Moreover, this behaviour was not similar within treatments and caused an interaction effect where the MI and urea treatments showed an increase toward day 15 and dropped again toward day 28, while the control and MI + urea showed constant values through all of the days. On the other hand, ADF content differed between treatments and was highest in the control (*p* < 0.0001; 33.5%), the lowest in urea (28.6%) and intermediate in MI and MI + urea (30.6%). Days of treatment increased (*p* < 0.0001) ADF content and reach an average value of 31.3% compared to 30.0% at day 1. The increase in ADF content behaviour differed between treatments during the ensiling days and caused an interaction effect (*p* < 0.0001). While in the control ADF content increased, in MI + urea it stayed stable. In the urea and MI treatments, it increased up to 15 days and then decreased toward day 28 (Figure S1). Hemicellulose content decreased (*p* < 0.0001; Table S1) during ensiling and was highest at day 1 (22.1%) and lower during the rest of the ensiling days (19.4%). Hemicellulose content was lowest (*p* < 0.0001) in the control (18.6%) compared to 21.0% for the rest. An interaction effect of treatment by days of ensiling existed similar to that for NDF and ADF. DM content decreased (*p* < 0.0001; Table S2) during ensiling, and at 28 days, it averaged 30.0%. Treatments had a significant effect on DM content (*p* < 0.0001), being lowest in the control, and interaction existed with a similar pattern as the for the cell wall carbohydrates.

### 3.8. Amino Acids Profile of Wheat and Corn Silages

The amino acids profile in wheat silage was similar between treatments (Table 9). However, methionine and lysine contents (expressed as a percentage of essential AAs; EAAs) were affected by additives. Methionine profile’s was the lowest in MI + urea and differed from the control, being 1.31% compared to 2.31% in the rest. On the other hand, lysine’s profile was lowest in the urea treatment compared with the other treatments (6.06 vs. 6.59%). Considering the AA measured, the profile of total AA (TAA) as a % of CP was similar among treatments. However, the MI + urea treatment, numerically, had a higher number compared to others.

Amino acids analysis for corn silage (Table 10) showed, in general, that additives caused a decrease in the profile of some essential AAs (EAAs) (lysine, histidine, valine and phenylalanine), with the lowest being consistently in MI + urea, and the variation between the control (highest) and MI + urea (lowest) ranged from 0.44 to 1.44% units; Table 10. A similar trend was also observed for some non-EAAs (NEAAs; serine, glutamic acid, glycine, tyrosine and aminobutyric acid) with a difference between 0.59 and 2.16% units. The profile of TAA in wheat silage tended (*p* < 0.053) to be lowest in MI + urea, highest in the control, and intermediate in the MI and urea treatments. When compared to the control, MI + urea differed and was significantly lower.

## 4. Discussion

Silage making is considered the best way to ensure the continuous supply of high-quality forage all year around in intensive farming systems such as dairy cows. Winter crops such as wheat and corn, as representatives of summer crops in Israel, are the main sources of high-quality roughage for dairy farming. Hence, in the current study, we investigated the effect of heterofermentative life culture additive (MI), urea and their combination on the quality and dynamic of fermentation. We further studied the effect of the above on aerobic stability, which is detrimental during the stage of feeding practices and on the quality of the CP (i.e., AAs profile).

### 4.1. Dry Matter of Forages and pH Value of Silages

The dry matter contents of wheat forage prior to ensiling were above the maximum value recommendation, and those of the corn forage were within the recommended range [25,26]. A 44% DM content in this study in Israel is considered high but can still be ensiled. A high DM content could be related to the later harvest relative to the stage of maturity (milk to dough [26]), or agronomical and weather circumstances. Ensilaging forage comprising a DM content lower than 25% may result in undesirable fermentation products [27], whereas a content higher than 50% is defined as difficult to ensile hindering efficient fermentation [25]. The DM contents of all silages were maintained within the original forages (Table S2). At 28 days, in wheat silage, the DM content in the urea treatment was higher than the control, and in corn silage, urea and MI + urea differed from the control. This could be attributed to a more efficient utilization of the nutrients when additives were involved during the fermentation process. Additionally, the supplements may contribute to DM content [28]. However, caution must be taken when considering DM content because this study was conducted in a laboratory setting, which may not mimic large scale silos or bunkers [29].

The pH level is one of the main indicators of silage quality and the success of anaerobic fermentation and whether there is sufficient organic acids’ production, mainly LA [29,30]. The acidic pH is responsible for preserving the silage by preventing the development of undesired microorganisms, which may lead to silage deterioration [31]. In all treatments for both corn and wheat silages at 28 days of ensiling, the pH values were below 4, which is desired for excellent preservation. This further indicates that both forages had enough water-soluble carbohydrates content and a weak buffering capacity [26]. However, in corn silage, a sharp drop in pH was observed after one day in fermentation and remained almost constant up to 28 days. These dynamics were similar to the results of both silages reported elsewhere [26]. In corn silage, the sharp drop in pH within one day could be attributed to the high-water-soluble carbohydrates that make corn easy to ensile [32,33]. The pH in the MI treatment was highest in corn and lowest in wheat silages compared to others at day 28. These differences between the two silages mimic the dynamics of the development of heterofermentative LAB in the MI treatment, the availability of water-soluble carbohydrates, and the production of LA and acetic acid, which become pronounced in the later stages of the fermentation process [26]. In heterofermentative additives, LA is converted to acetic acid and 1,2-propanediol [34], which might have affected the pH values depending on the fermentation dynamics. The higher pH values observed in the urea and MI + urea treatments at day 28 of fermentation in wheat silage are a reflection of the above-mentioned dynamics together with the effect of urea prolonging the fermentation duration, which could hamper the rapid decrease in pH [35].

It should be mentioned that the fermentation dynamics after 4 weeks of fermentation change and might affect the pH values of both silages in large-scale farming conditions [26].

### 4.2. Temperature Dynamics in Silages

Silage temperature is a combined reflection of the environment and microbial activity dynamics. It is believed that the temperature of silages stabilizes when fermentation matures and reaches a steady state where no further major microbial activities occurs and there are stable environmental conditions. Hence, the changes in silage temperatures herein reflect the differences between the forages, treatments and seasons (winter vs. summer).

Temperature in the control treatment of wheat silage dropped between day 7 and day 14 and remained at 21 °C, while other treatments with additives remained higher. The increase in temperature could be attributed to the increased activity of microorganisms that generate heat during the early stages of the fermentation [2,10]. A higher temperature in additive treatments (22 °C) relative to the control (21 °C) is still within the desirable optimal range (25–40 °C), bearing in mind the microorganisms have a wider temperature range to thrive of 5–50 °C [15,36]. The temperature was generally lower and almost level after 14 days of ensiling, which could be attributed to conditions that hamper microbial activity and stability [1]. This is vital in reducing the loss of nutrients such as proteins [2].

In corn silage (summer crop), the temperatures were highest after 1 day in ensiling and then reduced and stabilized. A stabilized temperature and silage maturation are essential to minimize nutrient losses as mentioned above. Despite having higher temperatures (28 °C), all treatments in corn silages were within the optimal range (25–40 °C) and similar to the temperatures in silos [2].

### 4.3. In Vitro Digestibility of Silages

In vitro experiments were conducted to evaluate the digestibility of DM and the NDF of the silages and give some comparative insight into the effect of additives on nutritional values. Cell wall carbohydrates digestibility is one of the parameters that may mirror the effect of the biological activity (hydrolysis and synthesis) of epiphytic and inoculated bacteria. In general, the in vitro digestibility for silages at 28 days in all treatments were within the range values summarized elsewhere [26,37].

During ensiling, additives decreased the in vitro digestibility of DM and NDF compared to the control in wheat silages, which had a mild increase. Different dynamics in digestibility values within treatments were observed with additives. During the first 4 weeks of ensiling, the dynamics of the biological processes are considered not to be stable and take between one and six months to stabilize [26]. However, at 28 days of ensiling, the *IV*DMD values were lower in the additive treatments, which may be as a result of the intensive fermentation process that utilized fermentable nutrients including hemicellulose hydrolyses, which converts into pentoses reducing the NDF content [1]. These results contradict with the findings of [37], where a comparison between *Lactobacilli* inoculum and a control did not show differences in the digestibility of wheat silage. The *IV*NDFD of wheat silage at 28 days was highest in MI + urea compared to other treatments, which implies a synergistic effect of supplying an available source of N that probably prompted the hydrolysis of hemicellulose as mentioned above and increased the digestibility of NDF.

For corn silages, MI did not affect *IV*DMD or *IV*NDFD in agreement with [37]. Looking at the day 28 results of both the urea and MI + urea treatments on *IV*DMD and *IV*NDFD gives an interesting deeper insight of the treatment effects on the dynamics of fermentation that occurred. Both treatments decreased the *IV*DMD, which emphasizes the utilization of available nutrients and furthers the addition of urea-supplied available N encouraging microbial growth and decreasing the overall ruminal digestibility of DM [38,39]. However, *IV*NDFD in these treatments increased by 24% compared to the control. This shows the effects of intensive fermentation in corn silage that occurred when supplying available nutrients (mainly N) and MI, which further hydrolyzed cell wall components and bonds to be readily available to ruminal microbes [28,37].

### 4.4. Cell Wall Carbohydrates’ Contents of Silages

The cell wall carbohydrates’ contents at 28 days of ensiling of wheat silage had minor differences among the treatments. However, the dynamics of hemicellulose content (as discussed above) indicated that the hydrolysis of NDF happened and released hemicellulose (up to day 7), and then was used to support bacterial fermentation and release organic acids, and thus lead to the loss of DM, which might mask the actual contents of cell wall components [31]. These dynamics happened in all treatments despite the anticipation that MI treatment would decrease NDF content by hydrolysis of the hemicellulose for better bacterial fermentation [25].

For corn silages, the dynamics and changes in ADF and hemicellulose contents were pronounced mainly at day 1 and 7 of ensiling. It was observed on day 1 that hemicellulose content was increased (released), especially in the urea treatment, and on day 7, all additives were higher than the control. This was followed by higher *IV*NDFD in both urea treatments. At day 28, urea and MI + urea had higher *IV*NDFD, while MI alone was a similar value to the control. This emphasizes the effect of urea on hydrolysis and the dissociation of cell wall carbohydrates as was suggested by [1,28]. On the other hand, urea might also improve enzymatic bio-hydrolysis by supplying readily available nitrogen to enhance fermentation as was discussed earlier. However, this claim was not supported by the profile of CP and AAs (see later discussion).

### 4.5. Lactic Acid and Volatile Fatty Acids in Silages

Lactic and VFAs are by-products of fermentation during ensiling, and are essential for preserving silages and decreasing the pH values. These are mainly acetic, propionic and butyric acids, which each have different effects on silages. Heterofermentative cultures (such as those in the MI supplement) ferment pentoses into lactic and acetic acid [40]. Acetic acid has a special interest because it possesses an antimycotic activity, that together with lactic acid prevents the development of fungi and molds under aerobic conditions (see later discussion).

In wheat silages, despite the absence of significant differences, total VFAs (mainly acetic acid) were 17% higher in all additive treatments compared to the control at 28 days of ensiling. Lactic acid increased by days and in all the additive treatments, and was highest in the MI treatment at 28 days, and led to the lowest pH. These results are in agreement with similar findings that showed that the pH of wheat forage that was ensiled with *Lactobacilli* cultures (homofermentative or heterofermentative) was lower than the control [41,42].

On the other hand, organic acids’ production in corn silages was affected by days of ensiling and treatments. Total VFAs’ production at 28 days was 70–90% higher among treatments compared to wheat silage, mainly because of acetic acid with the notable production of propionic and butyric acids. In the MI treatment, propionic acid was the highest, while acetic acid, total VFAs and lactic acid were the lowest. The latter were similar to the results of wheat silage. This could be as a result of the rapid proliferation of MI and the drop in pH causing a depression in the fermentation rate. Propionic acid, believed to be responsible for flavor [3], had values 10 times more in MI than in the other treatments. These results are similar to a study conducted by [43]. The addition of urea is recommended in energy-rich silage such as corn, enhancing fermentation depicted by a high lactic acid concentration [14]. Hence, both urea and MI + urea had higher total VFAs and lactic acid similar to [28,44] who showed that urea hindered the pH decline in corn silage despite an increase in lactic acid production.

That being said, it should be remembered that the production of lactic and acetic acids continues across days of fermentation and peaks after one or three months depending on the forage origin and maturity [26].

### 4.6. Aerobic Stability of Silages

Aerobic stability success is actually a multifunction expression of silage firmness, fermentation organic acids, other functional by-products and independent factors such as environmental temperature.

In general, the inclusion of MI in forages produced the most aerobically stable silages indicated mainly by lower CO_2_ production. In wheat silage, stability was confirmed by all parameters measured, e.g., lower pH value, CO_2_ production and molds’ CFU relative to other treatments. The least stable silages were the control and MI + urea. For corn silage on the other hand, all treatments had the same pH values and CFU of molds.

The inclusion of urea in silages compromised their aerobic stability. This might have happened by means of supplying a substrate to aerobic microorganisms and buffering capacity by supplying ammonium as a bio-hydrolysis by-product that might increase the pH levels and prompt mold activity [1,45]. Concentrations of lactic and acetic acids are considered the most pronounced factors to affect aerobic stability, especially when heterofermentative Lactobacilli inoculum is added to silages [3,30,46]. The MI additive is considered a heterofermentative inoculum, and from this point of view, our findings agree with several studies in which heterofermentative Lactobacilli added to wheat silages improved aerobic stability [41,46,47]. However, the concentration of acetic acid was lower in MI in both silages at 28 days of fermentation, suggesting that other factors might be involved in aerobic stability performance. According to Higa, the MI preparation contains additional microorganisms other than Lactobacilli, whose proliferation leads to the production of antioxidant substances [10,11]. Their amount and influence were not examined in this study. However, it is apparent that they might have played a major role in improving the aerobic stability in the MI treatments despite the lesser amounts of lactic and acetic acids in these treatments relative to the other treatments.

### 4.7. Crude Protein and Amino Acids’ Content in Silages

Crude protein and AA contents were measured to give a deeper insight on the effect of additives on true CP and NPN fraction. The AA analysis was performed after hydrolysis on dried silages at 28 days; thus, the results reflects both free and bound AA contents. That being said, it should be noted that most of the AA in silages are recovered in the free form [48,49]. In general, the dynamics by day of CP content in wheat silages during ensiling was the same within treatments; however, additives influenced the CP content and this was higher by 6% in urea and MI + urea than in the control and MI alone. However, this differed in the corn silages, and CP contents were influenced both by days of ensiling and treatments. At day 28 of ensiling, MI + urea had 56% more CP, and both MI and urea treatments had an extra 35% compared with the control. This phenomena can be explained by the differential dynamics of microbial activity in wheat and corn silages, which was very rapid in corn compared to wheat (rapid vs. slow drop in pH). A slower drop in pH during the first 7 days of ensiling might have allowed more endogenous plant and microbial enzymatic activity including urea and protein hydrolysis in wheat silages compared to corn [1,5,49]; hence, a lower CP content. Some of the plant enzymes function at a higher pH environment (pH 7–8; [49]). However, at later stages of ensiling when pH was lowest in both silages, there was an advantage for CP content in corn silage, probably because of intact urea and biosynthesis [1,45]. However, the latter was not supported by the profile of TAAs in corn silage.

Individual and total AA profiles in wheat silage were similar among treatments. The total AAs profile averaged 60% of CP, which is in line with other findings [48,49,50]. The profiles of lysine and methionine relative to total EAA were lowest in urea and MI + urea, respectively, which might imply the extensive metabolism (e.g., hydrolysis or/and synthesis) of microbial communities in silages [48]. The total AA content in corn silages was the lowest in the MI + urea treatment (52%) compared to 65% for others. Hence, the actual extra CP contents in the MI and urea treatments were not as a result of protein biosynthesis, confirming our previous conclusion related to the rapid fermentation effect. It further strengthens the conclusion that in MI + urea, the extra CP content originated from urea (NPN), which remained intact in corn silage. Moreover, the negative significant effect of additives on some of the essential and non-essential AA profiles in corn silage is in line with our conclusive understanding that there was no microbial biosynthesis of CP in silages as a result of additives. Whether there is an advantage of the effect of this extra CP on growth or production performances for ruminants is beyond the scope of this study, and remains yet to be determined in vivo.

## 5. Conclusions

In general, additives in this study improved the quality parameters of wheat and corn silages. This was achieved by improving the fermentation process and producing organic acids. The inclusion of MI produced the most aerobically stable silages, indicated mainly by lower CO_2_ production and pH values, especially in wheat silage. The inclusion of urea in silages compromised aerobic stability. Neither of the additives affected the true CP content of silage. However, a higher CP content was observed in all additive combinations in corn silage. This addition of NPN might be useful in ruminants’ rations; however, caution must be taken with access to N in high-producing ruminants. Additives improved *IV*NDFD in both silages and was the best when MI was combined with urea. This happened because of the partial hydrolysis of cell wall carbohydrates, namely hemicellulose, and was better pronounced in corn silage.

## Figures and Tables

**Table 1:** Chemical composition of the forage samples before ensiling on DM basis (except DM).

	pH	DM	OM	CP	NDF	ADF	Hemicellulose
Corn forage	5.50 ± 0.08	30.6 ± 0.48	95.7 ± 0.24	8.7 ± 0.65	52.2 ± 2.46	28.4 ± 1.62	23.9 ± 0.85
Wheat forage	6.49 ± 0.01	43.8 ± 0.25	93.3 ± 0.18	9.7 ± 0.45	57.2 ± 1.01	29.5 ± 0.54	27.7 ± 0.53

DM: dry matter; OM: organic matter; CP: crude protein; NDF: neutral detergent fiber; ADF: acid detergent fiber.

**Table 2:** The effect of additives and ensiling days on pH of wheat and corn silages.

**Wheat Silage**								
**Days**	**Treatment**					**Main Effect (*p*-Value)**		
	Control	MI	Urea	MI + Urea	SEM	Trt	Day	Trt × Day
1	6.64 ^b^	6.52 ^b^	6.97 ^a^**	6.99 ^a^**	0.078	<0.0001	<0.0001	<0.0001
7	4.33 ^a^	4.16 ^b^*	4.36 ^a^	4.41 ^a^	0.037			
14	3.96 ^ab^	3.81 ^b^	4.00 ^a^	4.02 ^a^	0.033			
28	3.84 ^c^	3.73 ^d^***	3.93 ^a^***	3.90 ^b^**	0.029			
**Corn Silage**								
**Days**	**Treatment**					**Main Effect (*p*-Value)**		
	Control	MI	Urea	MI + Urea	SEM	Trt	Day	Trt × Day
1	3.75	3.86	3.90	3.86	0.031	0.0047	0.0008	0.0399
7	3.62	3.68	3.65	3.73	0.021			
14	3.89	3.98	3.80	3.72	0.045			
28	3.62 ^b^	3.97 ^a^**	3.74 ^b^	3.68 ^b^	0.053			

LSMeans in rows with different superscripts differ, * 0.05, ** 0.01 and *** 0.001: statistical significance after LSMeans Dunnett test, SEM: standard error of the mean.

**Table 3:** The effect of additives and ensiling days on IVDMD (%) of wheat and corn silage.

**Wheat Silage**								
	**Treatment**					**Main Effect (*p*-Value)**		
**Days**	**Control**	**MI**	**Urea**	**MI + Urea**	**SEM**	**Trt**	**Day**	**Trt × Day**
1	57.2	60.2	54.0	55.7	1.001	0.0058	0.7763	0.0099
7	55.6	57.8	55.9	54.1	0.710			
14	59.0	54.7	57.0	53.7 *	0.840			
28	61.7 ^a^	54.2 ^ab^	52.6 ^b^*	56.8 ^ab^	1.436			
**Corn Silage**								
	**Treatment**					**Main Effect (*p*-Value)**		
**Days**	**Control**	**MI**	**Urea**	**MI + Urea**	**SEM**	**Trt**	**Day**	**Trt × Day**
1	60.8 ^a^	57.2 ^ab^	52.2 ^bc^**	48.6 ^c^**	1.804	<0.0001	0.1549	0.1328
7	60.4	54.1	49.9 *	50.2 *	1.838			
14	60.4 ^a^	61.0 ^a^	50.0 ^b^	47.1 ^b^	2.393			
28	55.8 ^ab^	56.7 ^a^	45.9 ^b^*	49.9 ^ab^	1.793			

LSMeans in rows with different superscripts differ, * 0.05, ** 0.01: statistical significance after LSMeans Dunnett test, SEM: standard error of the mean.

**Table 4:** The effect of additives and ensiling days on IVNDFD (%) of wheat and corn silage.

**Wheat Silage**								
	**Treatment**					**Main Effect (*p*-Value)**		
**Days**	**Control**	**MI**	**Urea**	**MI + Urea**	**SEM**	**Trt**	**Day**	**Trt × Day**
1	39.5 ^c^	48.1 ^a^***	43.2 ^b^*	45.1 ^ab^***	0.771	<0.0001	0.0014	<0.0001
7	39.9 ^b^	45.1 ^a^**	41.9 ^ab^	43.7 ^b^*	0.575			
14	40.6 ^b^	43.0 ^ab^	44.0 ^a^*	41.9 ^ab^	0.461			
28	40.3 ^b^	41.6 ^b^	39.9 ^b^	44.8 ^a^***	0.528			
**Corn Silage**								
	**Treatment**					**Main Effect (*p*-Value)**		
**Days**	**Control**	**MI**	**Urea**	**MI + Urea**	**SEM**	**Trt**	**Day**	**Trt × Day**
1	42.6 ^bc^	38.9 ^c^	45.2 ^ab^	47.5 ^a^*	0.807	<0.0001	0.0368	0.0008
7	43.4 ^ab^	43.2 ^b^	45.3 ^ab^	47.5 ^a^*	0.607			
14	40.0 ^b^	36.5 ^b^	48.3 ^a^***	45.2 ^a^*	0.990			
28	38.2 ^b^	40.3 ^b^	47.6 ^a^***	47.3 ^a^***	0.903			

LSMeans in rows with different superscripts differ, * 0.05, ** 0.01 and *** 0.001: statistical significance after LSMeans Dunnett test, SEM: standard error of the mean.

**Table 5:** The effect of additives and ensiling days on volatile fatty acids (g/100 g DM) of wheat silage.

Wheat Silage								
	Treatment							
	Control	MI	Urea	MI + Urea		Main Effect (*p*-Value)		
Days	Acetic Acid (g/100 g DM)				SEM	Trt	Day	Trt × Day
1	1.89	1.75	2.01	2.08	0.130	0.2098	0.6174	0.9963
7	1.98	1.84	2.18	2.27	0.084			
14	1.89	1.93	1.91	2.15	0.051			
28	1.89	2.07	2.24	2.31	0.118	Main Effect (*p*-Value)		
Days	Propionic Acid (g/100 g DM)				SEM	Trt	Day	Trt × Day
1	0.10	0.05	0.05	0.06	0.012	0.2001	0.0256	0.4722
7	0.04	0.04	0.04	0.01	0.008			
14	0.05	0.01	0.03	0.03	0.008			
28	0.02	0.02	0.04 *	0.04 *	0.004	Main Effect (*p*-Value)		
Days	Butyric Acid (g/100 g DM)				SEM	Trt	Day	Trt × Day
1	0.02	0.01	N.D	N.D	0.004	0.0513	0.0771	0.3841
7	N.D	N.D	N.D	N.D	0.000			
14	N.D	N.D	N.D	N.D	0.000			
28	N.D	N.D	N.D	N.D	0.000	Main Effect (*p*-Value)		
Days	Total Acid (g/100 g DM)				SEM	Trt	Day	Trt × Day
1	2.08	1.91	2.07	2.15	0.130	0.4212	0.8159	0.9945
7	2.11	1.95	2.22	2.28	0.081			
14	2.03	1.94	1.95	2.18	0.050			
28	1.91	2.09	2.28	2.35	0.120			

* 0.05: statistical significance at *p* < 0.05 after LSMeans Dunnett test, SEM: standard error of the mean, N.D: non-detectable.

**Table 6:** The effect of additives and ensiling days on volatile fatty acids (g/100 g DM) of corn silages.

Corn Silage								
	Treatment							
	Control	MI	Urea	MI + Urea		Main Effect (*p*-Value)		
Days	Acetic Acid (g/100 g DM)				SEM	Trt	Day	Trt × Day
1	1.59 ^b^	0.87 ^b^	2.50 ^a^*	2.86 ^a^*	0.300	<0.0001	<0.0001	0.1581
7	1.68 ^bc^	1.34 ^c^	2.96 ^ab^	3.45 ^a^*	0.346			
14	2.31 ^bc^	1.73 ^c^	3.00 ^ab^	3.60 ^a^*	0.274			
28	3.42	2.27	3.57	3.60	0.227	Main Effect (*p*-Value)		
Days	Propionic Acid (g/100 g DM)				SEM	Trt	Day	Trt × Day
1	0.02	0.16	0.02	0.02	0.027	<0.0001	0.0274	0.0328
7	0.01 ^b^	0.08 ^a^*	0.02 ^ab^	0.02 ^ab^	0.012			
14	0.02 ^b^	0.22 ^a^***	0.02 ^b^	0.01 ^b^	0.033			
28	0.03 ^b^	0.29 ^a^**	0.03 ^b^	0.02 ^b^	0.044	Main Effect (*p*-Value)		
Days	Butyric Acid (g/100 g DM)				SEM	Trt	Day	Trt × Day
1	0.08 ^ab^	0.05 ^b^	0.12 ^a^	0.11 ^a^	0.012	<0.0001	0.0122	0.3739
7	0.07	0.05	0.12	0.13	0.013			
14	0.10	0.05	0.23	0.14	0.028			
28	0.15 ^ab^	0.07 ^b^	0.18 ^a^	0.16 ^ab^	0.017	Main Effect (*p*-Value)		
Days	Total Acid (g/100 g DM)				SEM	Trt	Day	Trt × Day
1	1.68 ^bc^	1.09 ^c^	2.65 ^ab^*	3.12 ^a^*	0.308	<0.0001	<0.0001	0.2612
7	1.77 ^b^	1.47 ^b^	3.11 ^ab^	3.60 ^a^*	0.354			
14	2.44 ^bc^	2.01 ^c^	3.25 ^ab^	4.05 ^a^**	0.303			
28	3.60 ^ab^	2.63 ^b^	3.79 ^ab^	4.09 ^a^	0.226			

LSMeans in rows with different superscripts differ, * 0.05, ** 0.01 and *** 0.001: statistical significance after LSMeans Dunnett test, SEM: standard error of the mean.

**Table 7:** The effect of additives and ensiling days on aerobic stability of wheat and corn silages.

Wheat Silage								
	Treatment							
	Control	MI	Urea	MI + Urea		Main Effect (*p*-Value)		
Days	^1^ CO_2_ (g/kg DM)				SEM	Trt	Day	Trt × Day
14	24.1 ^a^	5.4 ^b^***	8.2 ^b^***	11.4 ^b^**	2.166	<0.0001	0.0217	0.0011
28	33.7 ^a^	3.4 ^b^***	40.4 ^a^	4.7 ^b^***	4.122			
	^2^ Sugar loss (g)							
14	16.4 ^a^	3.7 ^b^***	5.6 ^b^***	7.8 ^b^**	1.473	<0.0001	0.0217	0.0011
28	22.9 ^a^	2.3 ^b^***	27.5 ^a^	3.2 ^b^***	2.803			
	^3^ Molds (Log CFU g/DM)				SEM			
28	9.1	7.9 ***	8.2 ***	8.1 ***	0.112	<0.0001		
	pH				SEM			
28	7.1	3.9 ***	5.1 ***	4.1 ***	0.272	<0.0001		
Corn silage								
	CO_2_ (g/kg DM)				SEM	Trt	Day	Trt × Day
14	52.7 ^c^	37.2 ^d^***	71.2 ^a^***	63.5 ^b^**	3.273	<0.0001	<0.0001	0.3411
28	38.8 ^ab^	28.7 ^b^	54.5 ^a^	42.4 ^ab^	3.007			
	Sugar loss (g)				SEM			
14	35.5 ^c^	27.3 ^d^***	47.8 ^a^***	43.2 ^b^***	1.868	<0.0001	<0.0001	0.3208
28	26.4 ^ab^	20.4 ^b^	34.7 ^a^	35.5 ^a^	1.927			
	Molds (Log CFU g/DM)				SEM			
28	9.2	9.1	9.3	9.1	0.059	0.3948		
	pH				SEM			
28	4.3	4.1	3.9	3.7	0.097	0.1882		

^1^ The amount of carbon dioxide emitted from the silage during a 5-day exposure to air, ^2^ amount of sugar lost from the silages during the 5-day exposure to air and ^3^ Colony Forming Unit: CFU—a measure that expresses the number of mold populations that have developed in 1 g of dry matter; LSMeans in rows with different superscripts differ, ** 0.01 and *** 0.001: Statistical significance after LSMeans Dunnett test, SEM: standard error of the mean.

**Table 8:** The effect of additives and ensiling days on crude protein content (%) of wheat and corn silages.

**Wheat Silage**								
**Days**	**Treatment**					**Main Effect (*p*-Value)**		
	**Control**	**MI**	**Urea**	**MI + Urea**	**SEM**	**Trt**	**Day**	**Trt × Day**
1	9.02 ^bc^	8.81 ^c^	9.83 ^ab^*	9.89 ^a^*	0.152	0.0005	0.3004	0.2427
7	8.53 ^b^	9.25 ^ab^	9.66 ^a^**	9.60 ^a^**	0.144			
14	9.30	8.90	9.45	9.47	0.163			
28	9.17	9.73	9.60	9.80	0.133			
**Corn Silage**								
**Days**	**Treatment**					**Main Effect (*p*-Value)**		
	**Control**	**MI**	**Urea**	**MI + Urea**	**SEM**	**Trt**	**Day**	**Trt × Day**
1	8.33 ^b^	8.35 ^b^	10.8 ^a^***	10.5 ^a^***	0.318	<0.0001	<0.0001	<0.0001
7	8.28 ^c^	9.81 ^b^***	10.9 ^a^***	10.5 ^ab^***	0.281			
14	9.18 ^b^	9.28 ^b^	11.0 ^a^***	11.0 ^ac^***	0.255			
28	7.99 ^c^	10.5 ^b^***	11.1 ^b^***	12.5 ^a^***	0.420			

LSMeans in rows with different superscripts differ, * 0.05, ** 0.01 and *** 0.001: statistical significance at *p* < 0.05 after LSMeans Dunnett test, SEM: standard error of the mean.

**Table 9:** Amino acids composition (g/100 g CP) of wheat silage.

	Additive					
Amino Acid	Control	MI	Urea	MI + Urea	SEM	Main Effect (*p*-Value)
**Essential**						
Lysine	1.78	1.94	2.01	1.91	0.107	0.9385
Histidine	1.15	1.17	1.26	1.03	0.066	0.7777
Valine	2.31	3.29	4.04	3.31	0.286	0.1883
Phenylalanine	3.13	3.06	3.51	2.84	0.171	0.6873
Arginine	1.84	1.50	2.04	1.49	0.112	0.2126
Threonine	2.83	2.85	3.09	2.64	0.107	0.6365
Methionine	0.64	0.67	0.76	0.37	0.064	0.1307
Isoleucine	10.10	11.80	12.14	11.51	0.753	0.8641
Leucine	3.42	3.39	4.18	3.34	0.221	0.5864
**Non-essential**						
Serine	2.71	2.78	2.87	2.41	0.130	0.7342
Glutamic acid	9.95	8.84	10.71	9.34	0.405	0.4836
Glycine	3.66	3.95	4.09	4.04	0.106	0.6061
Tyrosine	1.77	1.93	2.20	2.00	0.121	0.7670
γ-Aminobutyric acid	1.38	1.48	1.25	1.61	0.073	0.4426
Proline	4.40	4.32	4.70	4.37	0.156	0.8209
Alanine	4.23	4.43	5.62	4.84	0.316	0.5081
Hydroxyproline	0.50	0.49	0.41	0.51	0.017	0.1235
Lysine% EAA ^1^	6.49 ^a^	6.56 ^a^	6.06 ^b^	6.71 ^a^	0.099	0.0480
Methionine% EAA ^2^	2.37 ^a^	2.27 ^a^	2.29 ^a^	1.31 ^b^*	0.172	0.0132
TEAA% CP ^3^	27.19	29.67	33.03	28.44	1.692	0.7537
TAA% CP	56.37	58.72	58.30	65.54	9.343	0.7815

Lysine% EAA ^1^: lysine expressed as a percentage of essential amino acids; Methionine% EAA ^2^: methionine expressed as a percentage of essential amino acids; TEAA% CP ^3^: total essential amino acids expressed as a percentage of crude protein; means within the same row with different superscripts differ; * 0.05: statistical significance after LSMeans Dunnett test, SEM: standard error of the mean.

**Table 10:** Amino acids’ composition (g/100 g CP) of corn silage.

	Additive					
Amino Acid	Control	MI	Urea	MI + Urea	SEM	Main Effect (*p*-Value)
**Essential**						
Lysine	2.56 ^a^	2.08 ^b^*	2.04 ^b^*	1.81 ^b^**	0.107	0.0118
Histidine	1.46 ^a^	1.38 ^a^	1.21 ^b^*	1.02 ^c^**	0.065	0.0028
Valine	4.42 ^a^	3.84 ^a^	3.97 ^a^	2.98 ^b^*	0.213	0.0326
Phenylalanine	3.96 ^a^	3.59 ^ab^	3.22 ^bc^*	2.72 ^c^**	0.181	0.0125
Arginine	2.27 ^a^	2.15 ^a^	1.85 ^ab^	1.64 ^b^	0.104	0.0509
Threonine	3.58	3.16	2.76	2.64	0.154	0.0617
Methionine	0.90	0.82	0.54	0.65	0.061	0.0792
Isoleucine	9.54	10.92	11.35	8.59	0.698	0.5852
Leucine	6.44	5.42	4.76	4.23	0.350	0.0764
**Non-essential**						
Serine	3.43 ^a^	2.78 ^b^*	2.90 ^b^*	2.62 ^b^**	0.020	0.0096
Glutamic acid	8.84 ^a^	8.29 ^a^	9.33 ^a^	6.68 ^b^*	0.407	0.0346
Glycine	4.66 ^a^	3.91 ^b^	4.05 ^ab^	3.36 ^b^*	0.188	0.0311
Tyrosine	2.17 ^a^	1.94 ^a^	1.90 ^a^	1.58 ^b^*	0.083	0.0217
γ-Aminobutyric acid	2.15 ^a^	1.57 ^b^	1.36b *	1.51 ^b^*	0.122	0.0309
Proline	4.82	4.39	4.25	3.75	0.162	0.0707
Alanine	7.65	6.67	5.90	5.70	0.319	0.0550
Hydroxyproline	0.63	0.48	0.48	0.43	0.033	0.0941
Lysine% EAA ^1^	7.32	6.27	6.45	6.88	0.205	0.2976
Methionine% EAA ^2^	2.58	2.47	1.69	2.49	0.160	0.1296
TEAA% CP ^3^	35.14	33.37	31.70	26.28	1.473	0.1288
TAA% CP	69.86	63.58	62.33	52.22 *	4.106	0.0533

Lysine% EAA ^1^: lysine expressed as a percentage of essential amino acids; Methionine% EAA ^2^: methionine expressed as a percentage of essential amino acids; TEAA% CP ^3^: total essential amino acids expressed as a percentage of crude protein; means within the same row with different superscripts differ; * 0.05 and ** 0.01: statistical significance at *p* < 0.05 after LSMeans Dunnett test, SEM: standard error of the mean.

## Data Availability

Supporting data of this research will be available on request from the corresponding author.

## Supplementary Materials

The following supporting information can be downloaded at: https://www.mdpi.com/article/10.3390/ani13132197/s1, Table S1: The effect of additives and ensiling days on hemicellulose content of wheat and corn silage, Table S2: The effect of additives and ensiling days on DM content of wheat and corn silage, Figure S1: (a) The NDF content of wheat silage with additives treatment at various days of ensiling. (b) The NDF content of corn silage with additives treatment at various days of ensiling. (c) The ADF content of wheat silage with additives treatment at various days of ensiling. (d) The ADF content of corn silage with additives treatment at various days of ensiling.
