# Supplementary material for: The Effect of Microbial Inoculum and Urea Supplements on Nutritive Value, Amino Acids Profile, Aerobic Stability and Digestibility of Wheat and Corn Silages
Source: Animals (Basel). 2023 Jul 4;13(13):2197. doi: 10.3390/ani13132197 (PMC10339903; doi:10.3390/ani13132197)
Supplement: Supplementary file 1 [file animals-13-02197-s001.zip › animals-2438309-supplementary.pdf]

Supplementary Materials:

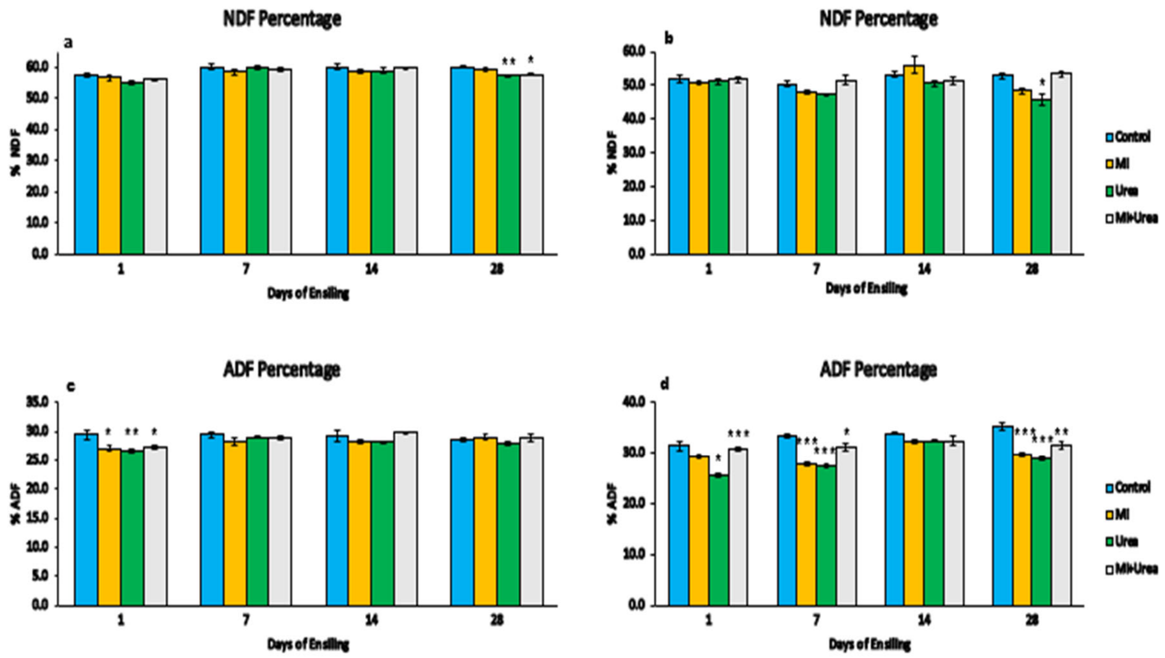

Figure S1. **a**. The NDF content of wheat silage with additives treatment at various days of ensiling; **b**. The NDF content of corn silage with additives treatment at various days of ensiling; **c**. The ADF content of wheat silage with additives treatment at various days of ensiling; **d**. The ADF content of corn silage with additives treatment at various days of ensiling. *0.05, **0.01 and ***0.001 Statistical significance at $P < 0.05$ after LSMeans Dunnett test.

Table S 1. The effect of additives and ensiling days on Hemicellulose content of wheat and corn silage

Wheat silage								
Days	Treatment					Main Effect (*P*-Value)		
	Control	MI	Urea	MI+Urea	SEM	Trt	Day	Trt*Day
1	29.4	29.5	28.5	28.9	0.258	0.0691	0.0029	0.4047
7	29.5	30.2	31.0	30.4	0.233			
14	29.1	30.7	30.6	29.9	0.354			
28	28.6	30.0*	29.4	28.9	0.205			
Corn silage								
Days	Treatment					Main Effect (*P*-Value)		
	Control	MI	Urea	MI+Urea	SEM	Trt	Day	Trt*Day
1	20.5	21.6	25.4**	20.9	0.604	0.0063	<.0001	0.0001
7	16.9	20.2**	20.0**	20.1**	0.436			
14	19.3	23.8	18.2	19.1	0.816			
28	17.6	18.6	16.8	21.9	0.726			

*0.05 and **0.01: Statistical significance at $p< 0.05$ after LSMeans Dunnett test, SEM: Standard error of the mean.

Table S 2: The effect of additives and ensiling days on DM content of wheat and corn silage

Wheat silage								
Days	Treatment					Main Effect (*P*-Value)		
	Control	MI	Urea	MI+Urea	SEM	Trt	Day	Trt*Day
1	43.8	45.0*	45.4*	43.2	0.346	<.0001	<.0001	0.0048
7	43.7	43.4	44.4	43.7	0.163			
14	43.9	43.0	44.6	43.4	0.279			
28	41.9	42.2	44.1**	42.7	0.332			
Corn silage								
Days	Treatment					Main Effect (*P*-Value)		
	Control	MI	Urea	MI+Urea	SEM	Trt	Day	Trt*Day
1	30.7	33.0	32.5	32.0	0.436	<.0001	<.0001	0.0290
7	29.6	31.9	31.6	30.6	0.463			
14	28.7	29.3	31.7**	30.8	0.500			
28	28.7	29.5	30.6*	31.0*	0.432			

*0.05, and **0.01: Statistical significance at $p < 0.05$ after LSMeans Dunnett test, SEM: Standard error of the mean.
